# Post-Catalytic Complexes with Emtricitabine or Stavudine and HIV-1 Reverse Transcriptase Reveal New Mechanistic Insights for Nucleotide Incorporation and Drug Resistance

**DOI:** 10.3390/molecules25204868

**Published:** 2020-10-21

**Authors:** Nicole Bertoletti, Albert H. Chan, Raymond F. Schinazi, Karen S. Anderson

**Affiliations:** 1 Department of Pharmacology, Yale University School of Medicine, New Haven, CT 06520-8066, USA; nicole.bertoletti@yale.edu (N.B.); alberthchan@gmail.com (A.H.C.); 2 Department of Molecular Biophysics and Biochemistry, Yale University School of Medicine, New Haven, CT 06520-8066, USA; rschina@emory.edu; 3 Center for AIDS Research, Laboratory of Biochemical Pharmacology, Department of Pediatrics, Emory University School of Medicine, Atlanta, GA 30322, USA

**Keywords:** nucleoside reverse transcriptase inhibitors, emtricitabine, stavudine, inhibitor-protein complexes, macromolecular X-ray crystallography

## Abstract

Human immunodeficiency virus 1 (HIV-1) infection is a global health issue since neither a cure nor a vaccine is available. However, the highly active antiretroviral therapy (HAART) has improved the life expectancy for patients with acquired immunodeficiency syndrome (AIDS). Nucleoside reverse transcriptase inhibitors (NRTIs) are in almost all HAART and target reverse transcriptase (RT), an essential enzyme for the virus. Even though NRTIs are highly effective, they have limitations caused by RT resistance. The main mechanisms of RT resistance to NRTIs are discrimination and excision. Understanding the molecular mechanisms for discrimination and excision are essential to develop more potent and selective NRTIs. Using protein X-ray crystallography, we determined the first crystal structure of RT in its post-catalytic state in complex with emtricitabine, (-)FTC or stavudine (d4T). Our structural studies provide the framework for understanding how RT discriminates between NRTIs and natural nucleotides, and for understanding the requirement of (-)FTC to undergo a conformation change for successful incorporation by RT. The crystal structure of RT in post-catalytic complex with d4T provides a “snapshot” for considering the possible mechanism of how RT develops resistance for d4T via excision. The findings reported herein will contribute to the development of next generation NRTIs.

## 1. Introduction

Since its discovery in the early 1980s, the human immunodeficiency virus 1 (HIV-1) has been a major health issue with nearly 38.0 million people infected globally in 2019 according to the WHO [1]. Despite extensive research efforts, neither a cure nor a vaccine for HIV-1 infection has been discovered yet. However, a breakthrough has been achieved with the highly active antiretroviral therapy (HAART), which significantly improves the life expectancy for patients with acquired immune deficiency syndrome (AIDS).

Two of the six classes of the United States Food and Drug Administration (FDA) approved drugs for HIV-1 treatment target the reverse transcriptase (RT) protein, an enzyme critical for the replication cycle of HIV-1 [2]. These two classes are nucleoside RT inhibitors (NRTIs) and non-nucleoside RT inhibitors (NNRTIs). While NNRTIs are allosteric inhibitors that alter the chemical catalysis rate limiting step through conformational changes [3,4], NRTIs mimic nucleotides that bind to the active site of RT. Since NRTIs lack the 3′-hydroxyl group necessary for chain elongation, their incorporation results in termination of the viral DNA transcription [5]. NRTIs are essential components of HAART and part of almost all FDA approved combination therapies for the treatment and protection of an infection with HIV. However, some FDA-approved NRTIs are now rarely prescribed (e.g., stavudine (d4T)) or discontinued (e.g., zalcitabine (ddC)) due to their off-target toxicity [6].

Several biochemical studies have examined NRTIs analogs by determining their incorporation efficiency (*k*_pol_/K_d_) relative to the respective natural deoxynucleotide triphosphates (dNTPs) [7,8,9,10,11]. Additionally, structural studies of HIV-1 RT by X-ray crystallography have elucidated the mechanism of reverse transcription initiation and incorporation of NRTIs. The combination of these studies has provided insight to how drug resistance to individual NRTIs develops [12,13,14,15,16,17,18,19,20,21]. Each NRTI acts as competitive inhibitor for the natural dNTP at the same binding site in RT; however, distinct mechanisms for discrimination and drug resistance have been observed. For instance, for emtricitabine ((-)FTC) the main mechanism of HIV-1 RT resistance is an altered discrimination, while for d4T, increased phosphorolytic excision of the incorporated monophosphate of the NRTI provides resistance [13,22,23,24]. Another limitation of NRTIs is the significant increase in HIV-1 RT resistance mutations upon treatment with different NRTI compounds [16,24,25]. A more detailed understanding of how various changes in the chemical structure of NRTI influence their ability to be incorporated and the structural consequences of interaction with RT will enable the development of more potent and selective NRTIs with a higher genetic barrier for acquiring drug resistance.

**Figure 1:**
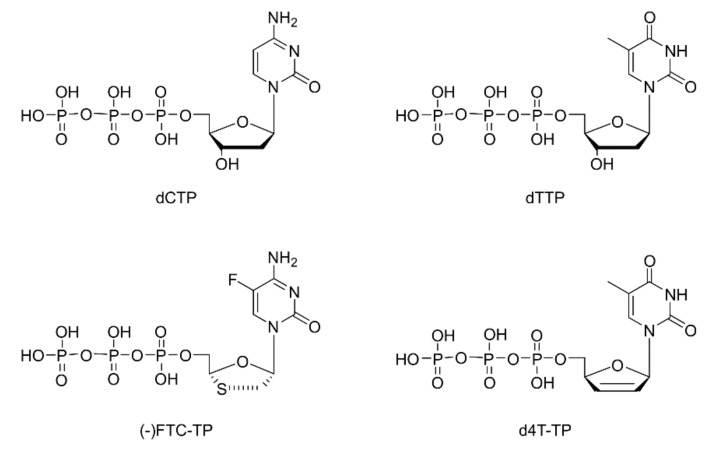
Chemical structures of 2′-deoxycytidine-5′-triphosphate (dCTP), and thymidine-5′-triphosphate (dTTP). (-)FTC-TP and d4T-TP are the triphosphate (TP) forms of emtricitabine ((-)FTC) and of stavudine (d4T), respectively.

In this study, we describe the crystal structures of RT in post-catalytic complexes with double stranded DNA (dsDNA) and two NRTIs (Figure 1)—the triphosphate form of cytidine analogue (-)FTC (2′,3′-dideoxy-5-fluoro-3′-thiacytidine) and the triphosphate form of the thymidine analogue d4T (2′,3′-didehydro-2′,3′-dideoxythymidine). The post-catalytic complex of (-)FTC monophosphate ((-)FTC-MP) incorporated into DNA primer of a dsDNA bound to RT, extends our earlier structural findings with the pre-catalytic complex. Together, these studies provide the structural framework for understanding the molecular mechanism of how this viral polymerase discriminates between (-)FTC and natural nucleotides [12], and the requirement to undergo a conformation change for successful incorporation by RT.

Comparing the crystal structure of RT in complex with d4T incorporated into the DNA primer and the crystal structure of RT in pre-catalytic complex with the triphosphate form of stavudine (d4T-TP) with a chain terminated dsDNA provides a “snapshot” on the possible mechanism of how RT develops resistance for d4T via excision. The results of this study together with previously presented data can contribute to the development of next generation NRTIs.

## 2. Results

Recombinant RT protein (Q258C and C280S mutations), crosslinked to the dsDNA primer/template (21-mer/27-mer) with an N2-cystamine 2′-deoxyguanosine six bases upstream from the priming site (P site), was used for the structural studies. A crystal structure of RT in complex with the DNA primer chain terminated with 2′,3′-dideoxyguanosine-5′-triphosphate (ddGTP) in the P site, and d4T-TP in the nucleotide-binding site (N site), was determined at a resolution of 2.74 Å (PDB ID: 6WPJ). A crystal structure of RT in complex with a DNA primer not chain terminated with deoxyguanosine triphosphate (dGTP) in the P site, and d4T monophosphate (d4T-MP) or (-)FTC monophosphate ((-)FTC-MP) incorporated in the DNA primer was determined at a resolution of 2.53 Å (PDB ID: 6WPF) and 2.72 Å (PDB ID 6WPH), respectively. Data processing and refinement statistics are listed in Table 1.

### 2.1. Determination of RT Crystal Structures in Complex with (-)FTC

**Figure 2:**
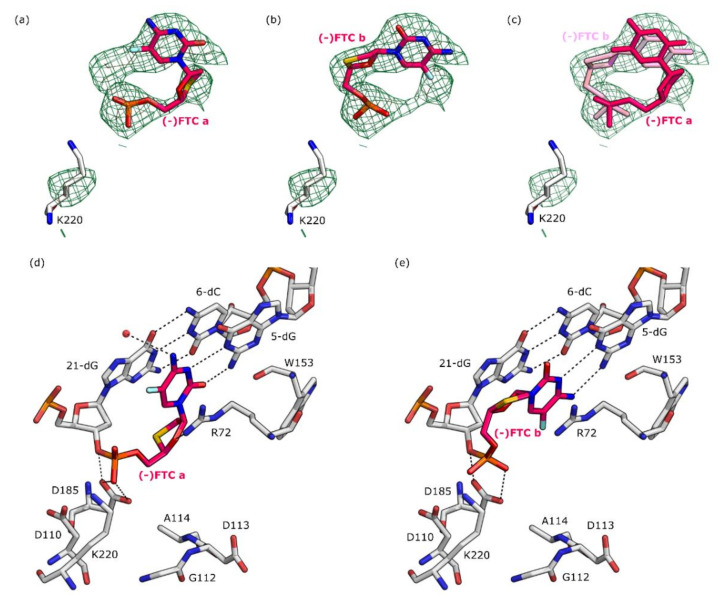
Crystal structures of RT in post-catalytic complex with dsDNA primer/template and (-)FTC (PDB ID: 6WPH). Amino acids and nucleic acids are shown as gray stick models. Hydrogen bonds (maximum distances 3.4 Å) with the (-)FTC-MP are depicted as black dotted lines. Water molecules are shown as red spheres. Carbon atoms of (-)FTC-MP are colored in magenta. A full list of hydrogen bond distances between the nucleoside reverse transcriptase inhibitors (NRTIs) and reverse transcriptase (RT) can be found in Figure S1. Panels (**a**,**d**): close-up view of the binding mode of copy A of (-)FTC-MP in the RT active site. Panels (**b**,**e**): close-up view of the binding mode of copy B of (-)FTC-MP in the RT active site. Panel (**c**): superimposition of the binding modes of both copies of (-)FTC-MP. In panels (**a**–**c**), the *F*_o_–*F*_c_ difference electron densities are shown as green mesh at a contour level of 3σ.

The overall structure of the post-catalytic complex of RT together with (-)FTC incorporated in the DNA primer does not highlight any significant changes compared to other structures of RT in complex with dsDNA and NRTIs. Residues 1–5, 66–67, 87–95, 212–232, and 429–452 in the p51 subunit and 134–141 in the p66 subunit were not included in the final model due to insufficiently defined *F*_o_–*F*_c_ difference electron density. The *F*_o_–*F*_c_ difference electron density in the N site reveals a double conformation of the incorporated (-)FTC-MP inhibitor (Figure 2). Copy A of (-)FTC-MP was refined to an occupancy of 40% and copy B of (-)FTC-MP was refined to an occupancy of 60%. The cytosine base moieties of both (-)FTC-MP copies maintain only two hydrogen bonds of the classical Watson–Crick base pair with 5-deoxyguanosine (5-dG) in the DNA template (Figure 2d,e). The α-phosphate groups of both copies are in hydrogen bond distance only with the side chain of residue D185. The copy A of (-)FTC-MP establishes further interactions, its primary amino group is in a long distance hydrogen bond (3.4 Å) with a water molecule, and its oxygen of the oxathiolane ring is interacting with the side chain of residue R72 (3.4 Å). No electron density of any Mg^+2^ ion is observed.

### 2.2. Determination of Crystal Structures of RT in Complex with d4T

**Figure 3:**
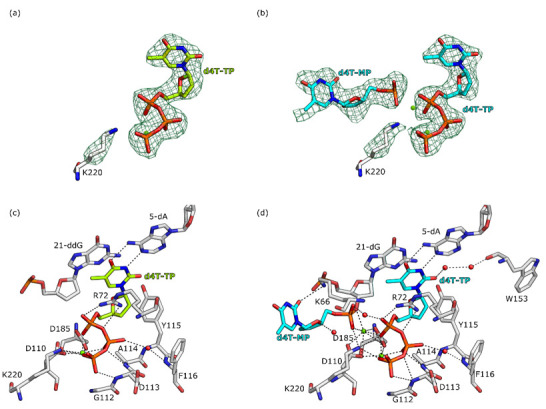
Close-up view of the NRTIs’ binding modes in the RT active site. Amino acids and nucleic acids are shown as gray stick models. Hydrogen bonds (maximum distances 3.4 Å) with the NRTIs are depicted as black dotted lines. Mg^+2^ ions are shown as green spheres. Water molecules are shown as red spheres. A full list of hydrogen bond distances between the NRTIs and RT can be found in Figure S2. Panels (**a**,**c**): structures of RT in complex with chain terminated DNA primer and d4T-TP at the N site (PBD ID: 6WPJ). Carbon atoms of d4T-TP are colored in light green. Panels (**b**,**d**): structures of RT in complex with d4T-MP incorporated in the DNA primer and d4T-TP at the N site (PDB ID: 6WPF). Carbon atoms of d4T-TP and d4T-MP are colored in cyan. Panels (**a**,**b**) show the *F*_o_–*F*_c_ difference electron densities as green mesh at a contour level of 3σ.

The overall structures of the two crystal structures of RT in complex with the inhibitor d4T are consistent with the previous structures of RT in complex with the dsDNA and the NRTIs [26,27]. For both crystal structures, the *F*_o_–*F*_c_ difference electron density at 3σ was not sufficient to build the amino acids 1–5, 87–95, 212–232, and 430–452 for the p51 subunit and 558–560 for the p66 subunit. In addition, for the structure of RT post-catalytic complex with d4T-MP incorporated in the DNA primer (PDB ID: 6WPF), no electron density was observed for amino acids 137 and 138 of the p66 subunit and therefore these residues were not included in the final model. In the structure with the chain terminated DNA primer (PDB ID: 6WPJ), a well-defined electron density for d4T-TP is observed in the N site of RT (Figure 3a). The thymine base moieties of the NRTI form Watson–Crick base pairs with the adenine base (5-dA) of the template DNA oligo and further form π-π stacking interactions with the guanine base 21-dideoxyguanosine (21-ddG) of the primer (Figure 3c and Figure S2). Only for the Mg^+2^ ion B (metal B in the polymerization mechanism [28]) the electron density was detected. Mg^+2^ ion B is highly coordinated with RT as well as with d4T-TP, achieving several interactions with the amino acid side chains of D110 and D185, the carbonyl of the backbone of V111, and the three phosphates group of d4T-TP. As previously described, the lack of a 3′-OH on the chain-terminated DNA primer results in a lower coordination number of the Mg^+2^ ion A to the complex (the Mg^+2^ ion corresponds to metal A in the polymerization mechanism). The lower coordination number results in a weaker binding of the Mg^+2^ ion A, leading to undetectable electron density [12,28]. The three phosphate groups are firmly anchored in the N site by forming several hydrogen bonds with the side chains of residues R72, D110, and D185, as well as with the backbone of residues V111, D113, and A114. The β-phosphate group is additionally stabilized by an indirect interaction with the backbone of residues Y115 and F116 via a water molecule (Figure 3c).

The structure of RT post-catalytic complex with d4T incorporated in the primer DNA (PDB ID: 6WPF) reveals the electron density of two copies of the inhibitor in the N site, one molecule of d4T-MP is incorporated in the DNA primer and is flipped out of the N site by a second molecule of d4T-TP (Figure 3b). The binding mode of the d4T-MP molecule incorporated in the DNA primer is stabilized by a long-distance hydrogen bond (3.2 Å) between its 2-keto group and the side chain of residue K66 (Figure 3d). Additionally, the oxygen in the 2,5-dihydrofuran moiety is in hydrogen bond distance with a water molecule (3.0 Å). The α-phosphate group of d4T-MP is in hydrogen bond distance with the side chain of residues D110 and K220 and is making an ionic interaction with the Mg^+2^ ion A. Furthermore, a water molecule is bridging an interaction between the α-phosphate group of the d4T-MP incorporated in the DNA primer and the α-phosphate group of d4T-TP in the N site. The Mg^+2^ ion B and d4T-TP achieve the same interaction as in the structure with the chain terminated dsDNA. Furthermore, Mg^+2^ ion B forms additional hydrogen bonds between the 2-keto group of the thymine based and side chain of residue Y115 and the backbone of residue W153 via water molecules. In the structure of the post-catalytic complex with d4T-MP incorporated in the DNA primer the electron density for Mg^+2^ ion A is well defined. The Mg^+2^ A ion is stabilized by several hydrogen bonds with the side chains of residues D110, D185, and α-phosphate group of both bound copies of the d4T molecule.

## 3. Discussion

### 3.1. Binding Mode Comparison of HIV-1 RT Complexes with (-)FTC

**Figure 4:**
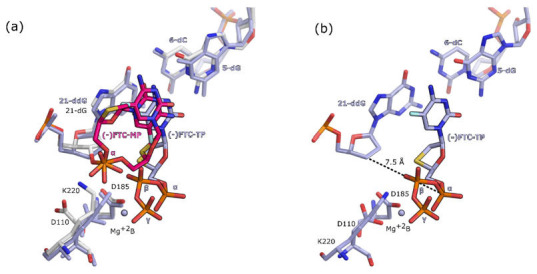
Close up view of the superimposition of binding pocket of the crystal structures of RT in complex with (-)FTC-MP incorporated in the DNA primer and the crystal structures of RT in complex (-)FTC-TP and chain terminated DNA primer with ddGTP. In panel (**a**), ligands (-)FTC-MP and (-)FTC-TP are shown as stick model. The carbon atoms of (-)FTC-TP, in the structure with chain terminated DNA primer (PDB ID: 6OR7 [12]), are colored violet, carbon atoms of (-)FTC-MP incorporated in the DNA primer (PDB ID: 6WPH), are colored magenta. The amino acids and nucleic acids are shown as stick models in gray for structure 6WPH and in violet for 6OR7. Mg^+2^ ions are shown as spheres colored in violet. In panel (**b**), close up view of the binding pocket of the crystal structures of RT in complex with (-)FTC-TP and chain terminated DNA primer with ddGTP. Distances are depicted as black dotted lines and given in Angstrom.

The overall superimposition of RT in complex with chain terminated DNA primer and (-)FTC-TP (PDB ID: 6OR7 [12]) and RT post-catalytic complex with (-)FTC-MP incorporated in the DNA primer (PDB ID: 6WPH) does not highlight significant differences. The RMSD of alignment of Cα atoms is 0.46 Å. The reported structure (Figure 4) shows that the 3′-OH of the preceding nucleotide is responsible for attacking the α-phosphate group of (-)FTC-TP. However, in the structure of RT post-catalytic complex with (-)FTC-TP, the α-phosphate group is located further away than the β-phosphate group from the preceding nucleotide (Figure 4b). These structural data suggest that conformational changes are necessary for successful incorporation of (-)FTC-TP by RT. This hypothesis is further supported by the two conformations that (-)FTC-MP adopts upon incorporation in the DNA primer. Moreover, previous pre-steady-kinetic studies reported the lack of a burst and a slower incorporation rates for (-)FTC-TP by HIV-1 RT (Table 2) [9,29]. This implies that the *k*_pol_ rate is similar or equal to the steady-state rate (*k*_ss_). These data, together with the structural data presented in this contribution, strongly suggest that conformational changes are necessary for the incorporation of (-)FTC-TP. It is further suggested that these conformational changes represent the slowest steps in the catalytic pathway. Moreover, the absence of electron density for Mg^2+^ ions indicates that (-)FTC is less susceptible to excision mechanism.

### 3.2. Binding Mode Comparison of HIV-1 RT Complexes with d4T 

**Figure 5:**
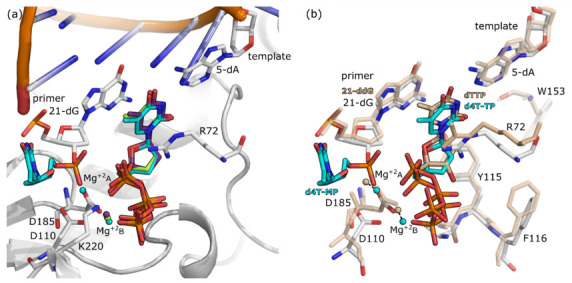
Superimposition of the binding pocket of the crystal structures of RT in complex with thymidine analogues. The amino acids and nucleic acids for RT in complex with d4T-MP incorporated in the DNA primer (PDB ID: 6WPF) are shown as gray stick models. Panel (**a**): superimposition of RT in complex with d4T-TP at pH 6.0 (PBD ID: 6WPJ), with d4T-TP at pH 9.5 (PBD ID: 6AMO [14]), and with d4T-MP incorporated in the DNA primer together with d4T-TP (PDB ID: 6WPF). Carbon atoms of d4T-TP at pH 6.0 are colored in light green. Carbon atoms of d4T-TP at pH 9.5 are colored in purple. Carbon atoms of d4T-MP incorporated in the DNA primer together with d4T-TP are colored in cyan. Panel (**b**): superimposition of RT in complex with dTTP (PBD ID: 1RTD [30]) and RT in complex with d4T-MP incorporated in the DNA primer. The amino acids and nucleic acids of RT in complex with dTTP are shown as beige stick models. Carbon atom of dTTP are color in beige. Mg^+2^ ions are shown as spheres color-coded as the ligand in their respective structures. Distances are depicted as black dotted lines.

The structure of the ternary complex of RT together with chain terminated dsDNA and d4T-TP inhibitor was previously elucidated by Martinez et al. [14]. However, it was reported that changes in the pH value during crystallization resulted in motion of the finger region [14]. Therefore, to be able to compare the binding modes of the different inhibitor under similar conformations of the protein, we reproduced this crystal structure at pH 6.0, the same pH as the other structure in this study. Alignment of the Cα atoms of the structures obtained at pH 9.5 (PDB ID: 6AMO [14]) and our structures obtained at pH 6.0 (PDB ID: 6WPF and 6WPJ) show an RMSD of 1.11 ± 0.03 Å, whereas structures obtained under the same pH of six show an RMSD of only 0.27 Å. Nevertheless, the binding mode of d4T-TP in the N site of RT is conserved in all the three structures (Figure 5a).

**Figure 6:**
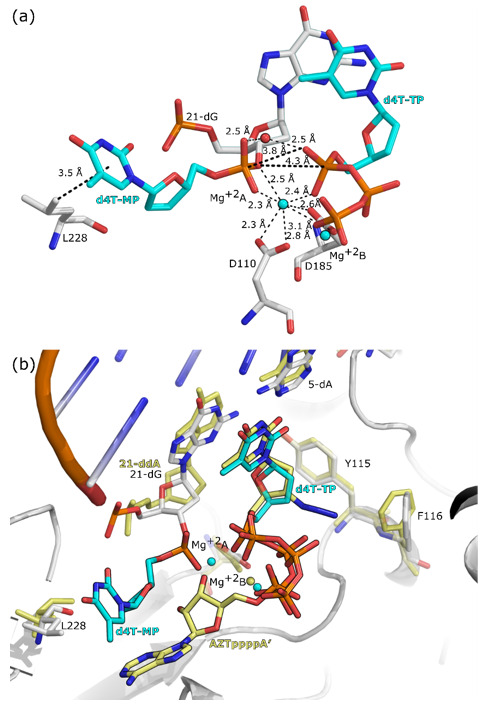
Close up view of the binding pocket of the crystal structures of RT in complex dsDNA primer/template, d4T-TP, d4T-MP, and AZTppppA’. Carbon atoms of d4T-TP, and d4T-MP are colored in cyan. Panel (**a**): structures of RT in complex with d4T-MP incorporated in the DNA primer and d4T-TP in the N site (PDB ID: 6WPF). Amino acids and nucleic acids are shown as gray stick models. Distances are depicted as black dotted lines. Mg^+2^ ions are shown as spheres colored in cyan. The water molecule is shown as a red sphere. Panel (**b**): superimposition of structures of RT in complex with d4T-MP incorporated in the DNA primer and the structure of RT in complex with AZTppppA’ (PDB ID: 3KLF [21]). Carbon atoms of AZTppppA’ are shown in yellow. Mg^+2^ ions are shown as spheres color-coded as the ligand in their respective structures. The amino acids and nucleic acids of the structure of RT in complex with AZTppppA’ are shown as yellow stick models. The amino acids and nucleic acids of the structure of RT in complex with d4T-MP incorporated in the DNA primer are shown as gray stick models.

The position of Mg^+2^ ion B is also conserved with small deviations of 0.2–0.3 Å between the three structures (Figure 5a). In contrast, the superimposition of the structure of RT in post-catalytic complex with d4T-MP incorporated in the DNA primer (PDB ID: 6WPF) with the structure of RT in complex with thymidine-5′-triphosphate (dTTP) (PBD ID: 1RTD [30]) shows a bigger shift of the positions of the Mg^+2^ ions. A shift of 1.2 Å between the Mg^+2^ ion As and of 1.1 Å between Mg^+2^ B ions is observed (Figure 5b). These variations are likely to be caused by the missing 3′-hydroxyl group of the chain terminated primer. In fact, the Mg^+2^ ion A in the structure of dTTP is less coordinated and only stabilized by three interactions with residues D110, D185, and the α-phosphate group of dTTP. In contrast, Mg^+2^ ion A achieves additional coordination with the α-phosphate groups of both d4T-MP and d4T-TP in the structure of RT in post-catalytic complex with d4T-MP incorporated in the DNA primer. A water molecule is bridging the two α-phosphate groups of d4T-MP and d4T-TP (Figure 6). This water molecule is potentially involved in the catalytic mechanism of incorporation/excision as proposed in previous studies [21].

Even though d4T-TP and dTTP have different structures, they are kinetically equivalent with respect to their incorporation efficiencies by HIV-1 RT (Table 2). Due to this similarity, the main mechanism of resistance for d4T is excision [24]. A mechanism of excision for zidovudine (AZT), ATP-mediated resistance that involves two Mg^2+^ ions, has been proposed based on structural results [21]. The excision reaction mechanism is the reverse of the incorporation of NRTIs, where the coordination of the Mg^2+^ ions for excision is similar to that for polymerization [21,31]. To elucidate the excision mechanism of HIV-1 RT for d4T, we compared the crystal structures of the AZT excision complex with the structure of RT in complex with d4T-MP and d4T-TP. Interestingly, the AZT triphosphate (AZT-TP) part of AZTppppA′ (a chemically synthesized excision product) and d4T-TP share a common binding mode (Figure 6b). The conserved binding mode of the phosphate groups of the AZT-TP part of AZTppppA′ and d4T-TP suggests that the excision mechanism of d4T is similar to the one described for AZT and, overall, the reverse of the catalytic mechanism of DNA polymerization. In fact, the α-phosphate group of d4T-TP is located ∼4 Å to the α-phosphate of d4T-MP, and the α-phosphate group of d4T-TP, acting as a nucleophile, could be attacking the α-phosphate of d4T-MP. Moreover, the crystal structure shows that d4T-MP is stabilized in the flipped conformation by van der Waals contacts with residue L228, which could facilitate the excision of d4T-MP from the primer.

## 4. Materials and Methods

### 4.1. Expression, Purification, RT-DNA Crosslinking and Crystallization of the Recombinant RT Protein

Recombinant RT enzyme containing the mutations C280S and Q258C was expressed in *Escherichia coli* strain BL21 (DE3) and purified as the following protocols previously described [12,27,32]. For the cross-linking experiments, a 27-mer DNA template (5′-ATGGGCGGCGCCCGAACAGGGACTGTG-3′ for the studies with (-)FTC and 5′-ATGGACGGCGCCCGAACAGGGACTGTG-3′ for the studies with d4T), standard desalted, was purchase from Integrated DNA Technologies (IDT) (Integrated DNA Technologies, Coralville, IA, USA) and a 20-mer DNA primer (5′-ACAGTCCCTGTTCGGXCGCC-3′) was purchased from TriLink Biotechnologies (San Diego, CA, USA). The cross-linking N2-cystamine 2′-deoxyguanosine in the DNA primer is indicated by an X. The cross-linking reaction between RT and 27-mer/20-mer primer/template was carried out as described by Sarafianos S.G. et al. [12,27,32]. Briefly, a solution containing 20 µM of annealed 27-mer/20-mer primer/template DNA and 10 µM of recombinant RT enzyme in 10 mM Tris, 25 mM NaCl, 10 mM MgCl_2_, and 25 µM dGTP set at pH 8 was incubated for 3 h at 37 °C. To obtain the complex with the chain terminated DNA (PDB ID: 6WPJ), a solution containing 20 µM of annealed 27-mer/20-mer primer/template DNA and 10 µM of recombinant RT enzyme in 10 mM Tris, 25 mM NaCl, 10 mM MgCl2, and 25 µM ddGTP (instead of dGTP) was incubated for 3 h at 37 °C. The complexes were then isolated as previously described [12]. Briefly, the complexes were purified using two affinity chromatography steps in tandem. The complexes were applied to a heparin column (5 mL HiTrap Heparin HP, GE Healthcare Life Sciences, Uppsala, Sweden) directly linked to a Ni-NTA column (5 mL HisTrap HP, GE Healthcare Life Sciences, Uppsala, Sweden). Unreacted RT protein bound to the heparin column, while the cross-linked RT-DNA complex passed through the heparin column and was subsequently trapped by the Ni-NTA column. After disconnecting the heparin column from the Ni-NTA column, the target protein was eluted from the Ni-NTA column by increasing the imidazole concentration in the buffer to 600 mM. The (-)FTC-TP was synthesized as previously described [29]. The purity of (-)FTC-TP compounds (~99%) was verified by high-pressure liquid chromatography analysis as well as liquid chromatography–electrospray ionization mass spectrometry. Crystals were prepared as previously reported by us [12]. In summary, equal volume of RT enzyme cross-linked complex (final concentration ~90 μM) was combined with a mother liquor of 4% (*w*/*v*) PEG 8000, 15 mM magnesium sulfate, 50 mM MES adjusted at pH 6.0 and 0.1 mM of either (-)FTC-TP or d4T-TP. After growing for two weeks at 22 °C, crystals were dehydrated in the mother liquor solution with increased PEG 8000 concentration by 5% each two hour to a final concentration of 35% and crystals were incubated overnight. Crystals were then harvested and exposed to a solution containing 35% (*w*/*v*) PEG 8000, 15 mM magnesium sulfate, 50 mM MES pH 6.0, 20% (*v*/*v*) glycerol and 0.1 mM of the triphosphate nucleoside analogues (d4T-TP or (-)FTC-TP) for 10 s and then flash frozen in liquid nitrogen.

### 4.2. Data Collection, Processing and Structure Determination and Refinement

Crystals of RT protein in complex with (-)FTC incorporated in the DNA primer (PDB ID: 6WPH), d4T incorporated in the DNA primer (PDB ID: 6WPF), and d4T-TP not incorporated (PDB ID: 6WPJ) were collected at NSLS-II FMX beam line and APS 24-ID-E beam line. The data collections were conducted at 0.98Å wavelength and at 100 K temperature on a silicon Dectris Eiger 16M detector. The data collected were indexed, processed and scaled with XDS [33]. Phases were obtained by molecular replacement with the program PHASER MR [34] from the CCP4 suite [35] using the structure of RT in complex with chain terminated DNA primer and (-)FTC-TP (PDB ID: 6OR7) as search model. A subset corresponding to 5% of all reflections were omitted during refinement and used for the calculation of R_free_. Model building was performed in COOT [36,37] and refinement using PHENIX.refine version 1.14-3260 [38]. (-)FTC-MP, d4T-MP and d4T-TP SMILE codes were created with Molinspiration v2018.10 [39]. Compounds coordinates and restraints were generated with the Grade Web Server [40] and eLBOW [41]. Cartesian simulated annealing, together with default parameters and secondary structure restraints were used as a first refinement step. Subsequently, refinement of XYZ coordinates and individual B-factors was alternated with structural adaptation in COOT until the model was readily built and provided the best possible explanation of the electron density, acceptable R-factors, geometry statistics, and Ramachandran statistics. TLS refinement was performed for all structures except for 6WPF with TLS groups selected from the TLSMD web server [42,43]. Crystallography programs were compiled by SBGrid [44]. Matthews coefficient and solvent content were calculated with Matthews_coef program from “CCP4 suite” version 7.0.053 [45,46]. Mean B factors were calculated with MOLEMAN [47]. Ramachandran plots were calculated with *MolProbity* [48]. RMSD were calculated with COOT [36,37]. All structural representations were prepared with PyMOL [49].

## 5. Conclusions

We present the first crystal structures of RT in post-catalytic complex with (-)FTC-MP and d4T-MP incorporated in the DNA primer and the crystal structure of RT in complex with d4T-TP. The RT post-catalytic complex with (-)FTC-MP evidenced that the 3′-OH of the preceding nucleotide is attacking the α-phosphate, validating our hypothesis that (-)FTC needs to undergo conformational changes in order to get successfully incorporated by RT. Furthermore, the RT post-catalytic complex with d4T reveals the electron density of two copies of the inhibitor. One copy of d4T-MP is incorporated in the DNA primer and is flipped out of the N site by a second molecule of d4T-TP bound in the N site. The comparison of the crystal structure of RT in the post-catalytic complex with d4T and the crystal structure of RT in pre-catalytic complex with d4T-TP provides a “snapshot” of the possible mechanism of how RT develops resistance to d4T via excision. The presented structures suggest that the excision mechanism of d4T is similar to the one described for AZT and, overall, the reverse of the catalytic mechanism of DNA polymerization. Taken together, this study will support the rationale for chemically modifying the d4T and (-)FTC backbones in order to design new NRTIs that minimize toxicity while retaining efficacy.

## Figures and Tables

**Table 1:** Data collection and refinement statistics.

PDB ID Code	6WPH: (-)FTC Complex	6WPF: d4T/d4T-TP Complex	6WPJ: d4T-TP Complex
Data collection and processing			
Wavelength (Å)	0.9793	0. 9792	0.9792
space group	C 2 2 2_1_	C 2 2 2_1_	C 2 2 2_1_
unit cell parameters *a, b, c,* (Å);	166.0, 168.1, 101.9;	165.6, 170.5, 103.3;	167.6, 170.1, 102.5;
Matthews coefficient (Å^3^/Da)	2.67	2.53	2.74
solvent content (%)	53.99	55.16	55.21
**Diffraction data**			
resolution range (Å)	30–2.72 (2.88–2.72)	50–2.53 (2.68–2.53)	50–2.73 (2.90–2.73)
unique reflections	37,728 (5620)	47,964 (6782)	38,882 (5942)
*R(I)sym* (%)	16.1 (97.7)	13.9 (98.9)	15.3 (125.7)
Wilson B factor (Å^2^)	64.91	46.2	62.72
completeness (%)	98.4 (90.9)	97.9 (87.1)	99.1 (94.8)
redundancy	25.3 (24.1)	7.2 (6.8)	7.2 (6.7)
*<I/σ(I)>*	20.33 (2.68)	13.4 (2.1)	12.46 (1.56)
CC(1/2) *	99.9 (91.1)	99.7 (76.8)	99.6 (67.3)
**Refinement**			
resolution range (Å)	29.52–2.72 (2.88–2.72)	47.68–2.53 (2.68–2.53)	47.56–2.73 (2.90–2.73)
reflections used in refinement (work/free)	37,728 (35,844/1884)	47,964 (45,565/2398)	38,882 (36,937/1945)
final R value for all reflections (work/free) (%)	19.7/25.7	18.2/23.1	20.3/25.6
protein residues (chain A/chain B)	548/391	555/394	557/394
dsDNA (primer/template)	18/22	18/23	18/23
water molecules	22	139	6
RMSD from ideality: bond lengths (Å)	0.008	0.007	0.007
RMSD from ideality: bond angles (°)	0.941	0.907	0.932
Mean *B* factor protein (Å^2^)	63.9	52.8	53.7
Mean *B* factor ligand (Å^2^)	83.7(A)/83.2(B)	46.5(d4T-TP)/69.4(d4T)	46.2(d4T-TP)
Mean *B* factor waters (Å^2^)	45.6	40.8	42.2
*Ramachandran plot*			
residues in most favored regions (%)	90.6	93.1	91.8
residues in additionally allowed regions (%)	9.4	6.9	8.2
residues in disallowed regions (%)	0	0	0

Values in parenthesis describe the highest resolution shell.

**Table 2:** Nucleotide and analog incorporation by RT.

DNA/DNA	Pyrimidine Analogs	*k*_pol_ (s^−1^)	*K*_d_ (µM)	*k*_pol_ (s^−1^)/*K*_d_ (µM^−1^)
D23/D45	(-)FTC-TP	0.039 ± 0.003 ^1^	12 ± 2 ^1^	0.0033 ^1^
D23/D45	dCTP	2.9 ± 0.2 ^2^	56 ± 9.8 ^2^	0.052 ^2^
D22/D45	dTTP	1.0 ± 0.1 ^3^	1.5 ± 0.5 ^3^	0.67 ^3^
D22/D45	d4T-TP	1.4 ± 0.1 ^3^	1.2 ± 0.3 ^3^	1.2 ^3^

^1^ Feng. J.Y. et al., [9]; ^2^ Ray. A.S. et al., [11]; ^3^ Vaccaro J.A. et al. [8].

## Supplementary Materials

The following are available online, Figure S1: Close-up view of (-)FTC’s binding modes in the RT active site; Figure S2: Close-up view of d4T-TP’s binding modes in the RT active site; Figure S3: Close-up view of d4T-TP’s binding modes in the RT active site.
