# Supplementary material for: Post-Catalytic Complexes with Emtricitabine or Stavudine and HIV-1 Reverse Transcriptase Reveal New Mechanistic Insights for Nucleotide Incorporation and Drug Resistance
Source: Molecules. 2020 Oct 21;25(20):4868. doi: 10.3390/molecules25204868 (PMC7587939; doi:10.3390/molecules25204868)
Supplement: Supplementary file 1 [file molecules-25-04868-s001.pdf]

Post-catalytic complexes with Emtricitabine or Stavudine and HIV-1 Reverse Transcriptase Reveal New Mechanistic Insights for Nucleotide Incorporation and Drug Resistance.

Nicole Bertoletti [1], Albert H. Chan [1], Raymond F. Schinazi [2,3] and Karen S. Anderson [1,*]

[1] Department of Pharmacology, Yale University School of Medicine, New Haven, CT 06520-8066, USA; nicole.bertoletti@yale.edu (N.B.); alberthchan@gmail.com (A.H.C.); karen.anderson@yale.edu (K.S.A.).

[2] Department of Molecular Biophysics and Biochemistry, Yale University School of Medicine, New Haven, CT 06520-8066, USA; rschina@emory.edu (R.F.S.).

[3] Center for AIDS Research, Laboratory of Biochemical Pharmacology, Department of Pediatrics, Emory University School of Medicine, Atlanta, GA 30322;

* Correspondence: karen.anderson@yale.edu; Tel.: +01-203-785-4526

Table of content:

- Figure S1: Close-up view of (-)FTC's binding modes in the RT active site.
- Figure S2: Close-up view of d4T-TP's binding modes in the RT active site.
- Figure S3: Close-up view of d4T-TP's binding modes in the RT active site.

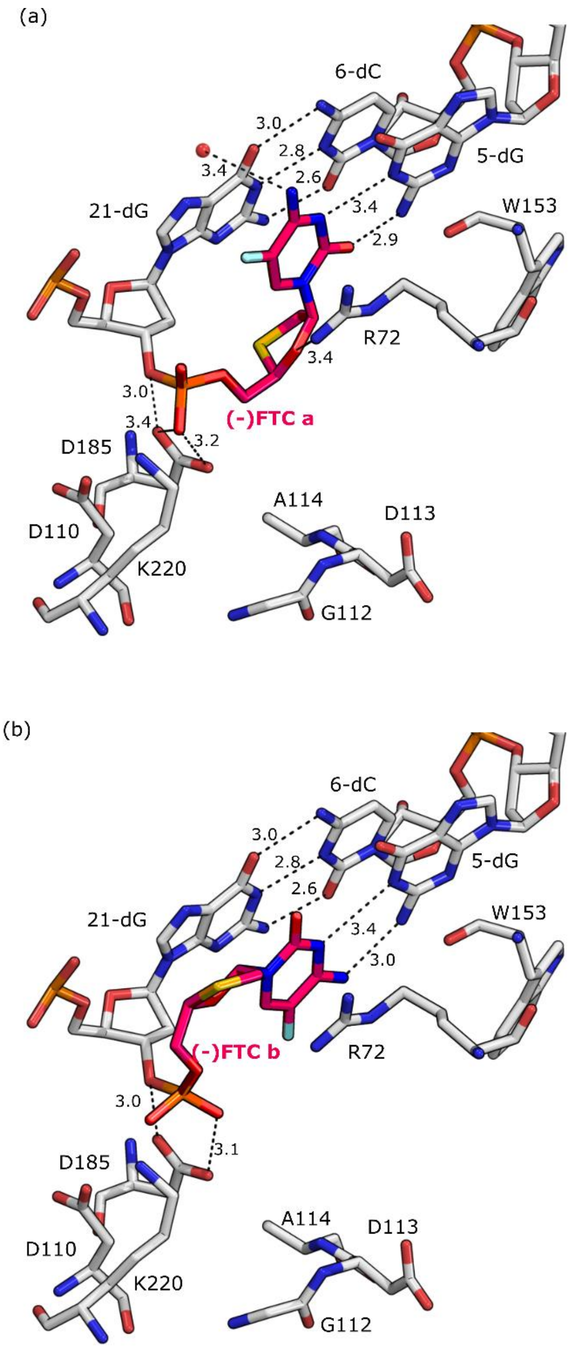

Figure S1. Close-up view of (-)FTC's binding modes in the RT active site (PDB ID: 6WPH). Amino acids and nucleic acids within 5 Å from the (-)FTC-MP are shown as gray stick models. Hydrogen bonds with the (-)FTC-MP are depicted as black dotted lines. Distances are given in Angstrom. Water molecules are shown as red spheres. Carbon atoms of (-)FTC-MP are colored in magenta. Panel (**a**): close-up view of the binding mode of copy A of (-)FTC-MP in the RT active site. Panel (**b**): close-up view of the binding mode of copy B of (-)FTC-MP in the RT active site.

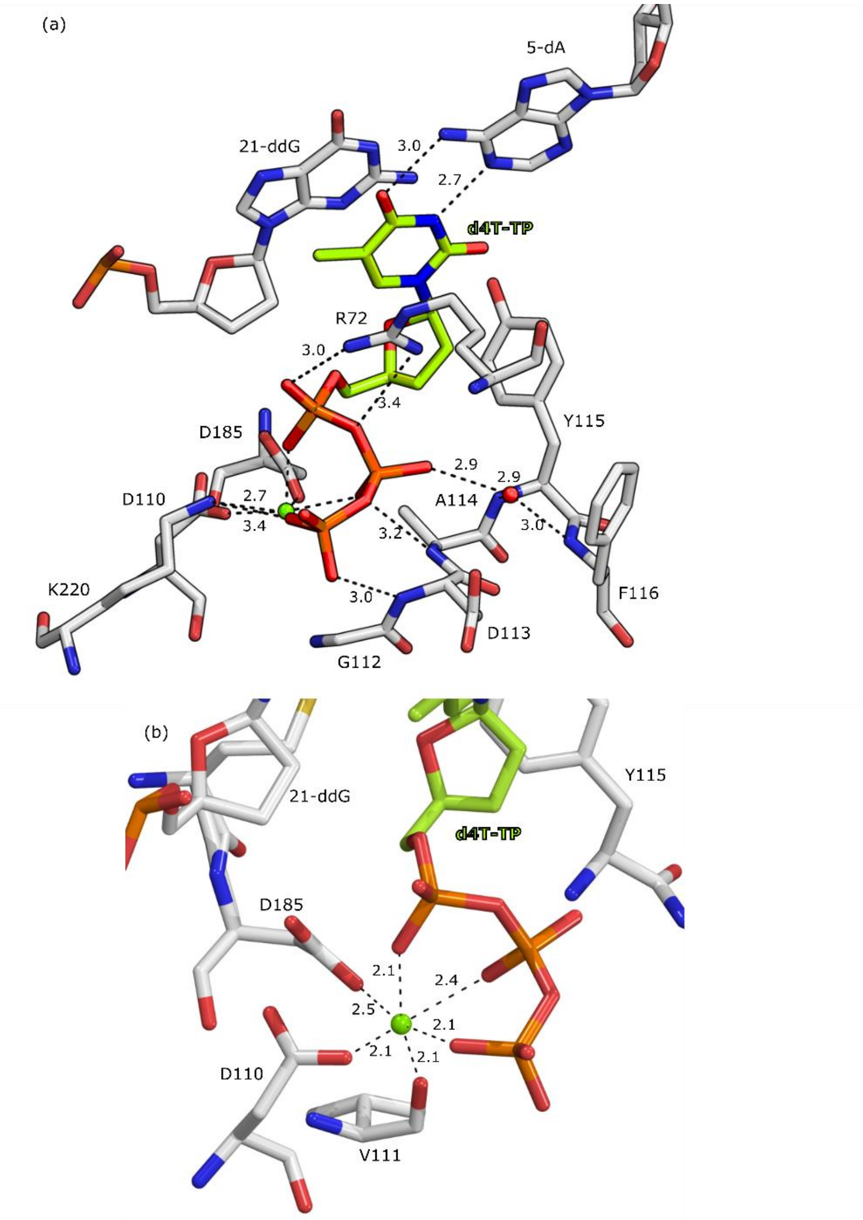

Figure S2. Close-up view of d4T-TP's binding modes in the RT active site. Amino acids and nucleic acids within 5 Å from the NRTIs are shown as gray stick models. Hydrogen bonds with the NRTIs are depicted as black dotted lines. Distances are given in Angstrom. Mg^{+2} ions are shown as green spheres. Water molecules are shown as red spheres. Panel (**a**): structures of RT in complex with chain terminated DNA primer and d4T-TP at the N site (PBD ID: 6WPJ). Carbon atoms of d4T-TP are colored in light green. Panel (**b**): close up view on the Mg^{+2} ions in the N site.

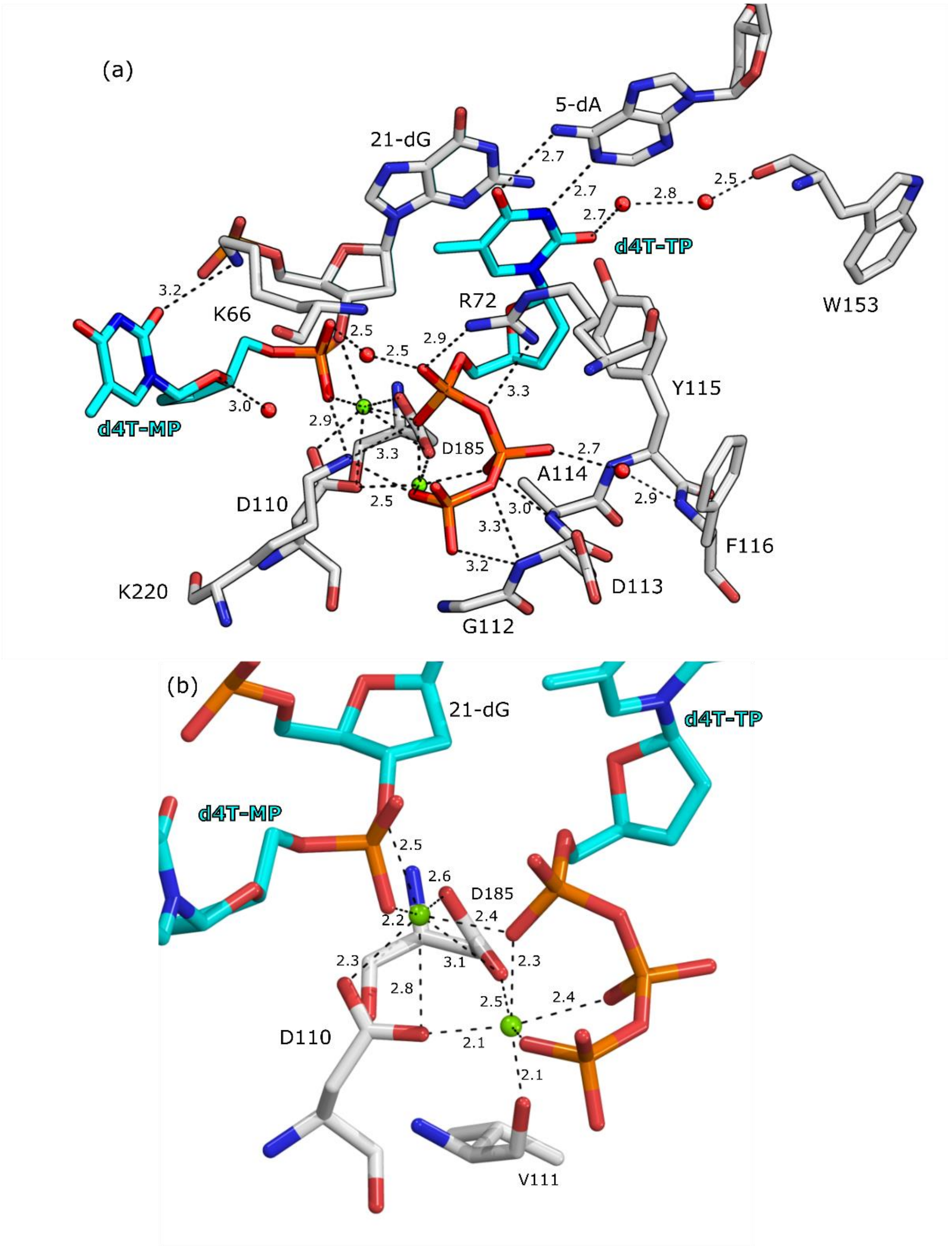

Figure S3. Close-up view of d4T-TP's binding modes in the RT active site. Amino acids and nucleic acids within 5 Å from the NRTIs are shown as gray stick models. Hydrogen bonds with the NRTIs are depicted as black dotted lines. Distances are given in Angstrom. Mg^{+2} ions are shown as green spheres. Water molecules are shown as red spheres. Panel (**a**): structures of RT in post-catalytic complex with d4T-MP incorporated in the DNA primer and d4T-TP at the N site (PDB ID: 6WPF). Carbon atoms of d4T-TP and d4T-MP are colored in cyan. Panel (**b**): close up view on the Mg^{+2} ions in the N site.
